# Glabellar and auricular leukemia cutis in CLL

**DOI:** 10.1002/jha2.538

**Published:** 2022-08-28

**Authors:** Bradley Steinman, Jose A. Plaza, Adam S. Kittai

**Affiliations:** ^1^ Ohio State University College of Medicine Columbus Ohio USA; ^2^ Department of Pathology and Dermatology The Ohio State University Columbus Ohio USA; ^3^ Department of Internal Medicine Division of Hematology The James Comprehensive Cancer Center The Ohio State University Columbus Ohio USA

1

A 68‐year‐old male with treatment‐naive chronic lymphocytic leukemia (CLL) presents with a right nasal bridge mass that was confirmed B‐cell leukemia on biopsy. He received localized radiation treatment and the mass resolved. Two years later, he developed an enlarging nasal bridge mass, frequent headaches, and new left glabellar and bilateral auricular masses in January 2022. The masses were firm, nontender skin‐colored nodules, the largest of which is on the left glabella. As per the patient, these lesions appeared and grew to their current size over 1–2 months. Laboratory testing demonstrated a white blood cell count of 115.74 × 10^9^/L (normal range: 3.73–10.10 × 10^9^/L) with an absolute lymphocyte count of 107.64 × 10^9^/L (normal range: 0.83–3.57 × 10^9^/L). Biopsy of the lesions shows a diffuse atypical monomorphic lymphoid proliferation in dermis and subcutaneous fat. The epidermis was uninvolved. The atypical monomorphic lymphocytes are small to medium sized with nuclear pleomorphism and cytologic atypia. These atypical lymphocytes are uniform and have scant cytoplasm and clumped chromatin. Prolymphocytes with dispersed chromatin and centrally placed prominent nucleolus were also noted (Figure 1). The atypical lymphocytes stained positive with CD20, Bcl‐2, CD43, and CD23 consistent with B‐cell lymphoproliferative disease. Peripheral blood immunophenotyping confirmed monoclonal B‐cell population that was positive for CD19, CD20, CD5, and CD23, and negative for CD10. The patient had a normal male karyotype, no abnormalities observed on fluorescent in situ hybridization, was immunoglobulin variable heavy chain mutated, and had no CLL‐related mutations detected on next generation sequencing. These findings confirmed the diagnosis of CLL. As leukemia cutis often signifies advanced‐stage disease, and the patient had significant symptoms related to his skin, systemic therapy to address the CLL was initiated with venetoclax plus obinutuzumab per CLL14 [1]. Following four cycles of treatment, all skin lesions resolved. Concurrently, the patient's laboratory testing showed improvements with a white blood cell count of 5.38 × 10^9^/L (normal range: 3.73–10.10 × 10^9^/L) and an absolute lymphocyte count of 1.81 × 10^9^/L (normal range: 0.83–3.57 × 10^9^/L) demonstrating systemic response to treatment.

**FIGURE 1:**
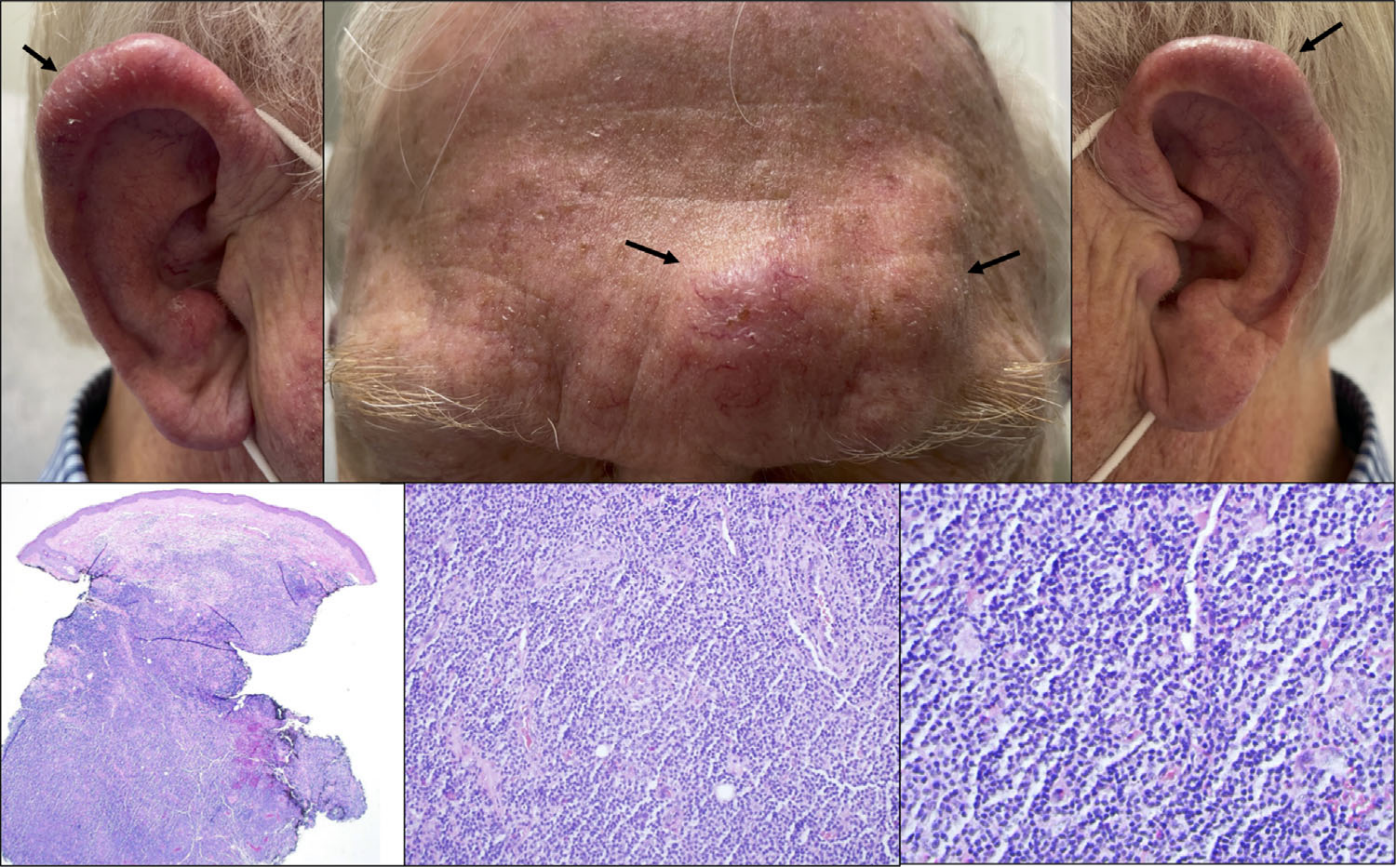
Glabellar (Top Middle) and Auricular (Top Left & Right) masses prior to initiating treatment. Low magnification biopsy (Bottom Left) shows a nodular to diffuse atypical lymphoid aggregate in dermis and subcutaneous fat. The epidermis is uninvolved. (H&E, 2x) High magnification biopsy (Bottom Middle, Right) shows small to medium‐sized lymphocytes with nuclear pleomorphism and cytologic atypia (H&E, 20x).

## CONFLICT OF INTEREST

A.S.K. receives research funding from AstraZeneca, and has consulted for Abbvie, AstraZeneca, Beigene, and Janssen. All other authors have no conflict of interest.

## FUNDING INFORMATION

The authors received no funding for this study.

## ETHICS STATEMENT

As per the Ohio State University institutional review board (IRB), no IRB protocol required for single case reports.

## PATIENT CONSENT STATEMENT

Patient has consented to use their photo and medical information in this publication. Signed consent available upon request.

## Data Availability

The authors confirm that data sharing is not applicable to this article as no datasets are generated or analyzed during the current study.
